# Cryo-EM structures of human fucosidase FucA1 reveal insight into substrate recognition and catalysis

**DOI:** 10.1016/j.str.2022.07.001

**Published:** 2022-10-06

**Authors:** Zachary Armstrong, Richard W. Meek, Liang Wu, James N. Blaza, Gideon J. Davies

**Affiliations:** 1 Department of Chemistry, Structural Biology Laboratory, University of York, Heslington, York YO10 5DD, UK; 2 The Rosalind Franklin Institute, Harwell Campus, Didcot OX11 0FA, UK

**Keywords:** glycobiology, carbohydrate active enzymes, fucosidase, glycans, fucosidosis, lysosomal storage diseases, FucA1, cryo-EM

## Abstract

Enzymatic hydrolysis of α-L-fucose from fucosylated glycoconjugates is consequential in bacterial infections and the neurodegenerative lysosomal storage disorder fucosidosis. Understanding human α-L-fucosidase catalysis, in an effort toward drug design, has been hindered by the absence of three-dimensional structural data for any animal fucosidase. Here, we have used cryoelectron microscopy (cryo-EM) to determine the structure of human lysosomal α-L-fucosidase (FucA1) in both an unliganded state and in complex with the inhibitor deoxyfuconojirimycin. These structures, determined at 2.49 Å resolution, reveal the homotetrameric structure of FucA1, the architecture of the catalytic center, and the location of both natural population variations and disease-causing mutations. Furthermore, this work has conclusively identified the hitherto contentious identity of the catalytic acid/base as aspartate-276, representing a shift from both the canonical glutamate acid/base residue and a previously proposed glutamate residue. These findings have furthered our understanding of how FucA1 functions in both health and disease.

## Highlights

• Cryo-EM structure of FucA1 resolves debate over the catalytic mechanism
• Aspartate 276, which is conserved for all animal homologs, is the catalytic acid/base
• A misfolded FucA1 disease variant (S150F) is stabilized by an iminosugar inhibitor

## Graphical abstract

**Figure:**
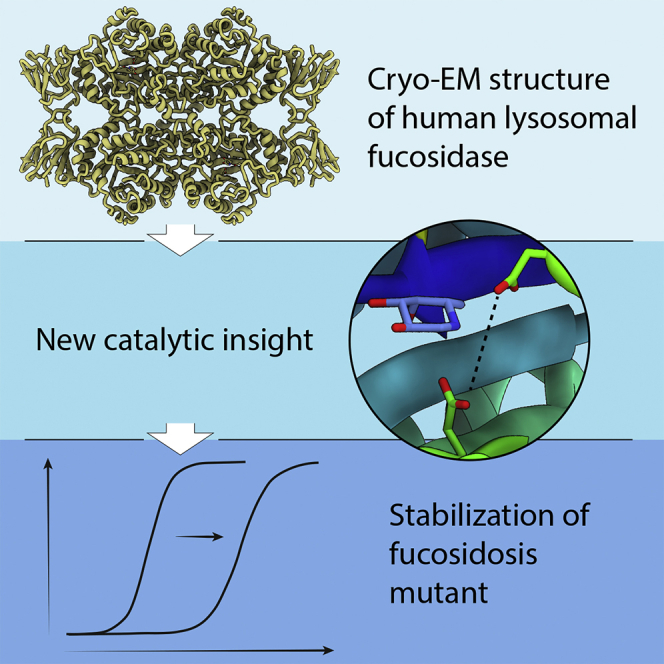

## Teaser

Human lysosomal fucosidase (FucA1) recycles fucosylated glycans. Aberrations in the enzyme result in fucosidosis. Armstrong et al. use single-particle cryo-EM to resolve the contentious mechanism of this enzyme. They also demonstrate stabilization of a misfolded variant by deoxyfuconojirimycin and determine how FucA1 accommodates this inhibitor.

## Introduction

Human N-linked glycans, ABO blood group antigens, Lewis antigens, keratan sulfate, epidermal growth factor (EGF)-like-domain-containing proteins, and even glycoRNA are decorated with α-L-fucose (Flynn et al., 2021; Varki et al., 2017). The presence or absence of fucosylated glycans has wide-ranging effects on processes that include antibody-dependent cellular toxicity (Falconer et al., 2018; Shields et al., 2002), lymphocyte development (Liang et al., 2018, 2019), angiogenesis (Wang et al., 2017), fertilization (Pang et al., 2011), cell adhesion (Li et al., 2018), and host-microbiome interactions (Kashyap et al., 2013). In addition, an altered presence of fucosylated glycans has been observed in several pathological processes, including cancer progression (Barthel et al., 2009; Nakamori et al., 1993) and viral infection (Bournazos et al., 2021; Larsen et al., 2021). In humans, the dynamic process of α-L-fucose removal is catalyzed by two α-L-fucosidases—lysosomal FucA1 and secreted FucA2—both of which liberate α-L-fucose from fucosylated glycoconjugates. Human fucosidases have been implicated in regulating bacterial infection (Liu et al., 2009), while FucA1 mutations can lead to α-L-fucosidosis, a debilitating neurodegenerative lysosomal storage disorder (Stepien et al., 2020). Understanding the structure and catalytic mechanism of human α-L-fucosidases is central to understanding their function.

Both human α-L-fucosidases belong to glycoside hydrolase family GH29 (http://www.cazy.org/) (Lombard et al., 2014). This enzyme family catalyses hydrolysis with a net retention of stereochemical configuration at the anomeric center (Sulzenbacher et al., 2004). The catalytic mechanism for GH29s relies on a pair of carboxylic-acid-containing residues, with one acting as a nucleophile and the other acting as a Brønstead acid/base. Previous work detailing the structural and biochemical characterization of GH29s has revealed that although the catalytic nucleophile (an aspartate) is conserved across this family, the catalytic acid/base is not conserved (Shaikh et al., 2013). The identity of the catalytic acid/base varies between the two subfamilies (A and B) found within GH29. Subfamily B employs a subfamily conserved glutamate as an acid/base residue (Shaikh et al., 2013). The acid/base residue for subfamily A enzymes (which includes both human fucosidases) is also thought to be a glutamate; however, its position is not conserved within the subfamily, as evidenced by structural studies on bacterial enzymes from *Paenibacillus thiaminolyticus* and *Thermotoga maritima* (Kovalová et al., 2019; Sulzenbacher et al., 2004). To further complicate matters, the catalytic acid/base for FucA1 has been proposed by Liu et al. (2008) to be glutamate-289. This residue aligns with a solvent-inaccessible glutamate-281 in *T. maritima* that is remote from the active site in bacterial subfamily A enzymes (Sulzenbacher et al., 2004) and thereby ill positioned for catalysis. Structural understanding of FucA1 will help to resolve the catalytic mechanism of this enzyme.

Herein, we describe the structure of FucA1 as determined by single-particle electron cryo-microscopy (cryo-EM). This has enabled detailed insight into FucA1 tertiary and quaternary structures and the structural basis for several fucosidosis causing mutations. Furthermore, a 2.49 Å resolution structure of FucA1 bound to the inhibitor deoxyfuconojirimycin has allowed us to identify, unequivocally, the enzyme residues vital to catalysis. Crucially, we have conclusively identified the proton donor used by this enzyme as Asp276. This is an unexpected result as all GH29s were thought to employ a glutamate as an acid/base and Asp276 had not previously been predicted to play a catalytic role. We also show that the catalytic aspartate acid/base is conserved for all animal GH29 fucosidases, resolving an important, long-standing uncertainty in the field (Klontz et al., 2020; Lammerts van Bueren et al., 2010a, Lammerts van Bueren et al., 2010b, Shaikh et al., 2013).

## Results and discussion

### Cryo-EM structure reveals a homotetrametric structure

*FUCA1* has previously been expressed in *E. coli* (Liu et al., 2008). However, as this protein is naturally glycosylated (Alhadeff et al., 1975), we chose to express it in eukaryotic *Trichoplusa ni* (that typically forms N-glycans with paucimannosidic structures [Shi and Jarvis, 2007]) using the baculoviral expression vector system (Vijayachandran et al., 2014). *FUCA1* was expressed with an N-terminal melittin secretion sequence followed by a TEV cleavable hexahistidine tag. FucA1 has previously been reported as a tetramer with an estimated mass of 220–230 kDa (Alhadeff et al., 1975). We confirmed tetramerization for our construct by size-exclusion chromatography with multi-angle laser light scattering (SEC-MALLS), which indicated a solution-state mass of 225 kDa (Figure S1).

**Figure 1:**
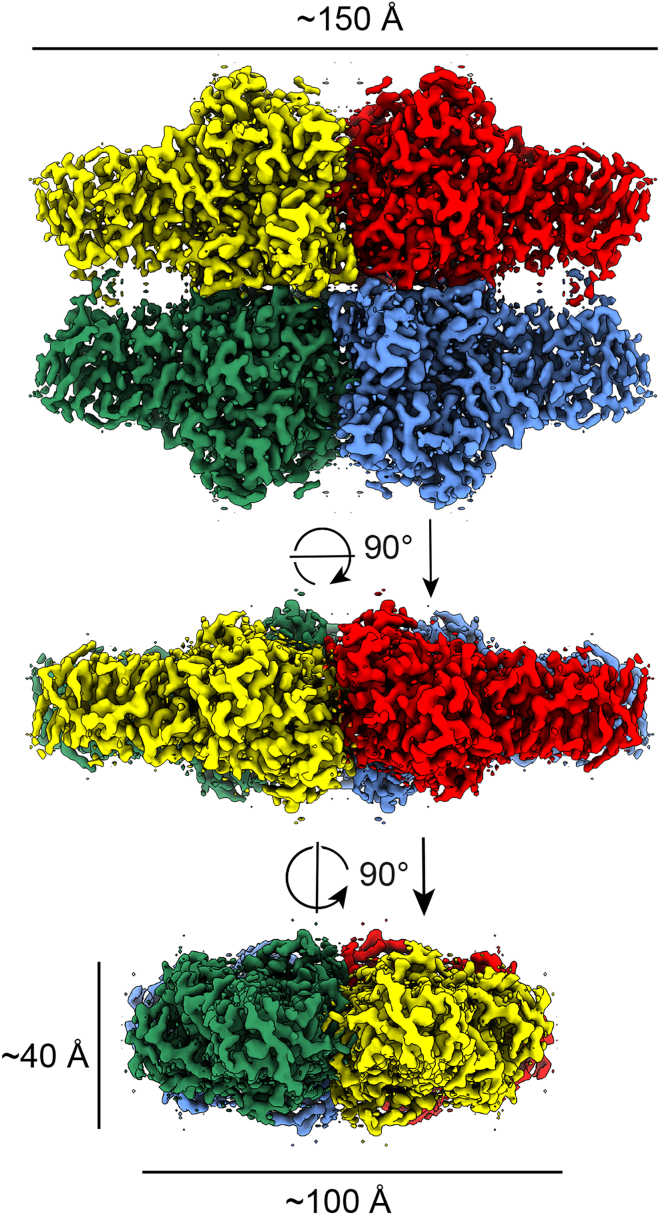
Cryo-EM structure of FucA1 Single-particle cryo-EM reconstruction of the FucA1 tetramer showing front and side views. Map is contoured at a threshold of 0.032, and individual protomers are colored in yellow, red, blue, and green.

To determine the structure of FucA1, we performed single-particle cryo-EM using a 200 kV Thermo Fisher Scientific Glacios electron cryo-microscope equipped with a Falcon-4 detector. Micrographs showed particles with diverse angular coverage, and secondary structural features could be observed in two-dimensional (2D) class averages (Figure S2). Further data processing resulted in a 3D reconstruction of FucA1 with dihedral symmetry (D2) and an overall resolution of 2.49 Å (see Figure S2 and Table 1 for data collection and refinement statistics). FucA1 forms a symmetric homotetramer approximately 150 × 100 × 40 Å in size (Figure 1). The map quality was sufficient to build residues 31–461 of each protomer, with a resulting map-model correlation of Fourier shell correlation (FSC) = 0.5 at 2.5 Å (Figures S2–S4).

**Table 1:** Cryo-EM data collection, refinement, and validation statistics

	FucA1 unliganded (EMDB-13499) (PDB: 7PLS)	FucA1-DFJ(EMDB-13520) (PDB: 7PM4)
**Data collection and processing**		

Nominal magnification	150,000	240,000
Voltage (kV)	200	200
Electron exposure (e−/Å^2^)	50	50
Defocus range (μm)	−1.2, −1.5, −1.8	−1.2, −1.5, −1.8
Pixel size (Å)	0.934	0.574
Symmetry imposed	D2	D2
Initial particle images (no.)	941,836	277,456
Final particle images (no.)	171,847	98,815
Map resolution (Å) (FSC threshold)	2.49 (0.143)	2.49 (0.143)

**Refinement**		

Initial model used (PDB)	N/A	N/A
Model resolution (Å) (FSC threshold)	2.53 (0.5)	2.51 (0.5)
Model resolution range (Å)	2.45–3.28	2.45–3.53
Map sharpening *B* factor (Å^2^)	−24.8	−42.05
Model composition		
Non-hydrogen atoms	14,224	14,288
Protein residues	1,724	1,724
Ligands	4	8
*B* factors (Å^2^)		
Protein	41	37
Ligand	41	32
RMSDs		
Bond lengths (Å)	0.004	0.004
Bond angles (°)	0.968	0.95
Validation		
MolProbity score	1.71	1.61
Clashscore	4.66	4.75
Poor rotamers (%)	1.33	1.06
Ramachandran plot		
Disallowed (%)	0	0
Allowed (%)	5.59	4.90
Favored (%)	94.41	95.10

**Figure 2:**
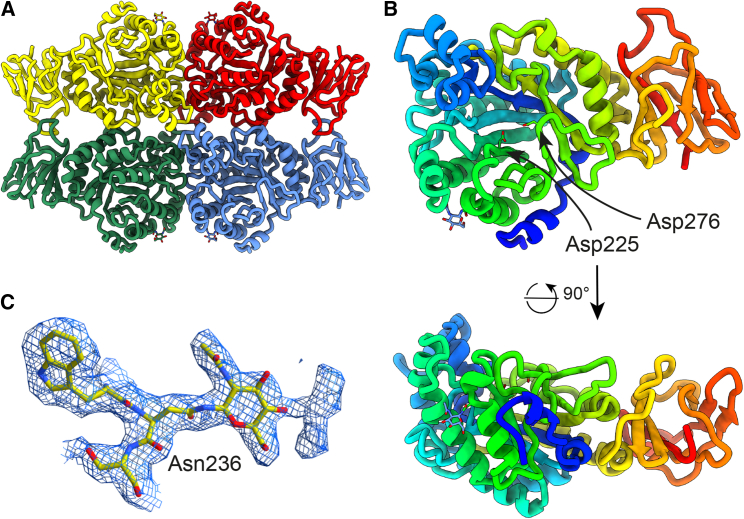
Tertiary and quaternary structures of FucA1 (A) The quaternary structure of FucA1 in cartoon representation. Each protomer is given a different color. N-glycans are shown in stick format. (B) FucA1 protomer in cartoon representation and colored from the N terminus in violet to the C terminus in red. Active-site residues Asp225 and Asp276 are shown in stick format. Front and side views are shown. (C) Glycan present on asparagine 236 of unliganded FucA1. Blue mesh is the density map at a threshold of 0.028. A weaker density could be observed for the second GlcNAc of the core chitobiose, but it was of insufficient quality to model.

Each protomer of FucA1 is comprised of an N-terminal catalytic domain—a modified (β/α)_8_ barrel—and a C-terminal β-sandwich domain (Figures 2A and 2B), which are characteristic of GH29 enzymes (Lombard et al., 2014). Each protomer interacts with two other protomers through residues in both the catalytic and β-sandwich domains, including the loop between residues 441 and 451, thus creating a flattened quaternary structure. A homo-multimeric quaternary structure has also been observed for other GH29 family members, including *P. thiaminolyticus* α-L-fucosidase isoenzyme 1 (PDB: 6GN6, hexameric [Kovalová et al., 2019]), *T. maritima* α-L-fucosidase (PDB: 1HL9, hexameric [Sulzenbacher et al., 2004]), and *Lactobacillus casei* AlfC (PDB: 6O18, tetrameric [Klontz et al., 2020]); however, the interaction interfaces are not conserved within this family. We also observed cryo-EM density corresponding to one of the three predicted glycosylation sites. A single N-acetylglucosamine could be built into density at position Asn236, which is near the interface between two of the catalytic domains (Figure 2C).

### Active-site structure reveals D276 as the catalytic acid/base

**Figure 3:**
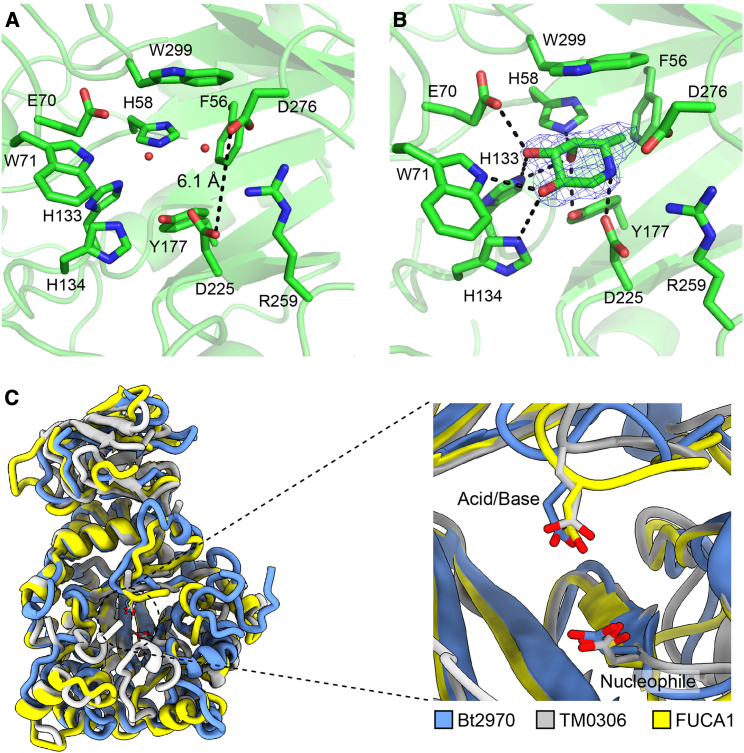
The catalytic center of FucA1 (A) Active site of unliganded FucA1. The distance between the catalytic acid and nucleophile is shown as a black dashed line. (B) Interactions between DFJ and the active site of FucA1. Polar interactions within hydrogen-bonding distance (3.2 Å) are shown as dashed lines. The density surrounding DFJ is at a threshold of 0.026. (C) Superposition of bacterial GH29 fucosidases onto FucA1. The bacterial fucosidases TM0306 (PDB:1HLl8 [Sulzenbacher et al., 2004], shown in white) and Bt2970 (PDB: 2XIB [Lammerts van Bueren et al., 2010b], shown in blue) are superimposed on FucA1, which is shown in yellow. The nucleophilic aspartate residues (D225, D224, and D229 for FucA1, TM0306, and Bt2970, respectively) and acid/base residues (D276, E266, and E288 for FucA1, TM0306, and Bt2970, respectively) overlap for all 3 structures.

To understand substrate binding and the active center of catalysis, we determined the structure of FucA1 in complex with the nanomolar iminosugar inhibitor deoxyfuconojirimycin (DFJ) (Winchester et al., 1990). This resulted in a cryo-EM reconstruction with nearly the same resolution as the uncomplexed form of FucA1 (2.49 Å, FSC threshold = 0.143; Figure S3). Cryo-EM density corresponding to the presence of DFJ could clearly be observed in the active site of FucA1 (Figures 3B and S5). The FucA1-DFJ complex revealed DFJ in a ^1^*C*_4_ conformation (φ: 16.7°, θ: 176.0°), consistent with previous complexes between GH29 fucosidases and DFJ (Lammerts van Bueren et al., 2010b; Sakurama et al., 2012) and representing a conformation expected for the Michaelis complex (Lammerts van Bueren et al., 2010a). Accommodation of DFJ did not result in substantial changes to the positioning of active-site residues (root-mean-square deviation [RMSD] of 0.2 Å for a sphere of amino acids 8 Å from DFJ; Figures 3 and S5). Within the active site, DFJ makes hydrogen-bond interactions with 7 amino acids, including the GH29-conserved nucleophile Asp225, positioned 2.8 Å from the endocyclic nitrogen of DFJ (Figure 3B).

**Figure 4:**
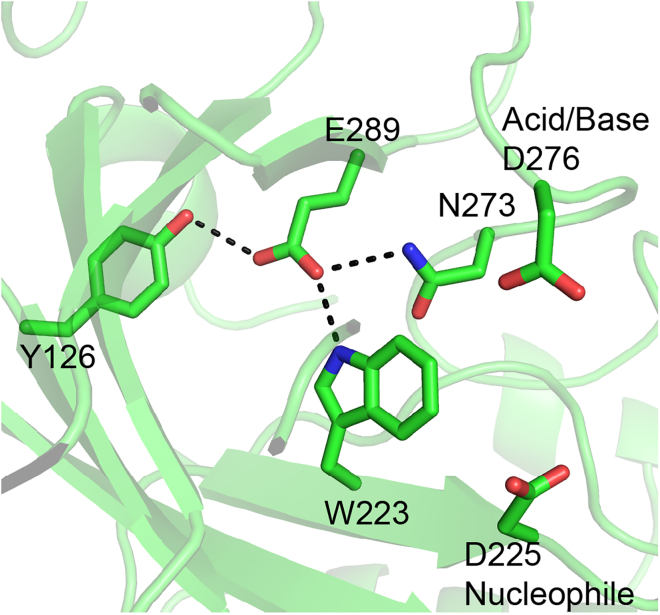
Positioning of Glu289 adjacent to the catalytic core Black dashes indicate interactions between Glu289 and adjacent residues that are within hydrogen-bonding distances. The catalytic acid/base and nucleophile do not interact directly with Glu289 and are shown for context.

Unlike the aspartate nucleophile, which is conserved across all GH29s, the identity of the catalytic acid/base varies between the two subfamilies (classes A and B) of GH29 (Shaikh et al., 2013). The catalytic acid/base for class-B enzymes—corresponding to Glu288 from *B. thetaiotaomicron* (Lammerts van Bueren et al., 2010b)—is conserved within this subfamily (Figure S6). The acid/base for class-A GH29s (which includes FucA1) is also considered a glutamate; however, its position is not conserved, as determined by structural studies of bacterial enzymes (Kovalová et al., 2019; Sulzenbacher et al., 2004). Furthermore, none of the structurally determined acid/base residues align with Glu289 proposed by Liu et al. for FucA1 (Liu et al., 2008). In the structure of FucA1 determined here, Glu289 is distant from the active site (10.5 Å from the catalytic nucleophile), positioned on the 7^th^ β-strand of the (α/β)_8_ barrel, precluding its presence on a flexible loop (Figure 4), indicating that it cannot be the catalytic acid/base of FucA1.

The FucA1-DFJ-complex structure reveals the positioning of Asp276 to be 3.4 Å from the endocyclic nitrogen, and 5.8 Å from the catalytic nucleophile (Figure 3B), a distance that is expected for a retaining glycoside hydrolase. Asp276 occupies a position equivalent to that of the acid/base of α-fucosidases from both *T. maritima* (Sulzenbacher et al., 2004) and *B. thetaiotaomicron* (Lammerts van Bueren et al., 2010b), belonging to GH29 classes A and B, respectively (Figure 3C), thereby supporting the role of Asp276 as the catalytic acid/base of FucA1. To date, this is the only observation of an aspartate acid/base within GH29 fucosidases. This shift from the canonical glutamate acid/base is associated with a repositioning of the acid/base loop (residues 274–288) closer to the active site, thereby compensating for the shorter side-chain length of aspartate (Figure 3C).

To further confirm our annotation of Asp276 as the genuine acid/base, we performed Michaelis-Menten kinetics with both FucA1 and an Asp276Asn variant, with both the activated substrate pNP-α-L-fucose (pNP-FUC) and fucose-α-1,2-galactose, a component of blood group antigens. In both cases, Asp276Asn had a substantially decreased specificity constant (Table 2; Figure S7); furthermore, the *K*_M_ for hydrolysis of pNP-FUC by the acid/base variant was lower, likely reflecting an accumulation of fucosyl-enzyme intermediates. We also re-evaluated the activity of the Glu289Gln variant, initially proposed by Liu et al. to be the catalytic acid/base. Interestingly, in our hands, the activity of this variant on pNP-FUC was below the limit of detection (Table 1). Wondering if this was a result of the introduced mutation causing substantial misfolding/unfolding, we used circular dichroism (CD) to check the secondary structure and found that although slightly different, the spectra did not drastically deviate from that of the wild-type enzyme, suggesting that the protein is folded (Figure S8).

**Table 2:** Kinetic parameters for fucosidase activity

Enzyme	Substrate	k_cat_ (s^−1^)	K_*M*_ (mM)	k_cat_/K_*M*_ (s^−1^, M^−1^)
FucA1-WT	pNP-α-L-Fuc	9.96 ± 0.09	0.41 ± 0.02	24,000 ± 1,000
FucA1-D276N	pNP-α-L-Fuc	0.059 ± 0.001	0.10 ± 0.02	600 ± 100
FucA1-WT	Fuc-α-1,2-Gal	0.74 ± 0.02	1.12 ± 0.08	660 ± 30
FucA1-D276N	Fuc-α-1,2-Gal	-	-	<20^a^
FucA1-E289Q	pNP-α-L-Fuc	-	-	<2^b^
FucA1-G60D	pNP-α-L-Fuc	-	-	<2^b^
FucA1-S150F	pNP-α-L-Fuc	0.713 ± 0.006	0.45 ± 0.02	1,550 ± 60

a Estimated from the limit of fucose detection.

b Estimated from the limit of pNP detection.

**Figure 5:**
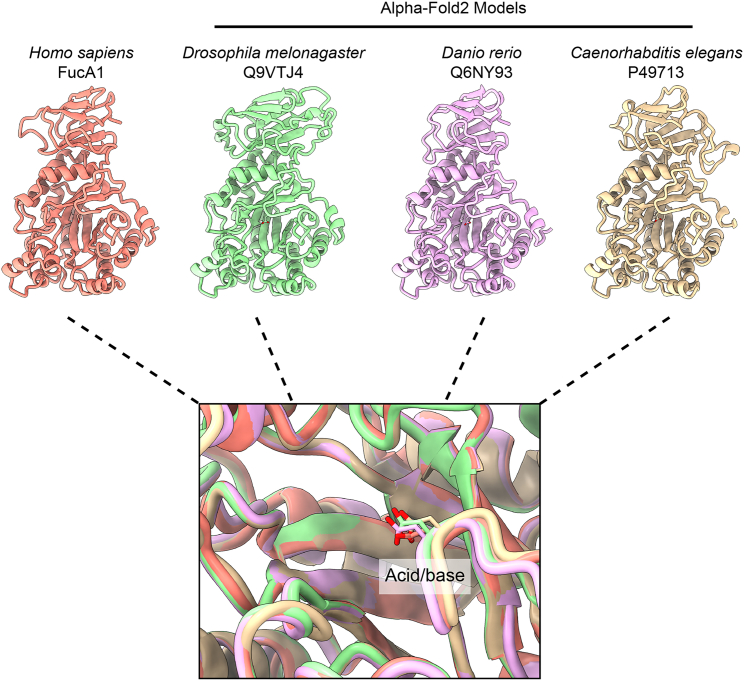
Superposition of the predicted structures of animal GH29 fucosidases and FucA1 Structural predictions for the fucosidases from *D. melonagaster*, *D. rerio*, and *C. elegans* were taken from the AlphaFold structural database (Jumper et al., 2021). The conserved acid/base residues overlap for all 4 structures.

The identity of the true aspartate acid/base residue is conserved for animal GH29s. Within subfamily A, the animal GH29s form a sub-branch, and a multiple sequence alignment of these enzymes shows that aspartate-276 is conserved in essentiallyall animal fucosidases (Figure S9). We also sought to examine this conservation through the structural alignment of animal fucosidases present in the AlphaFold database (Jumper et al., 2021). Alignment of AlphaFold-predicted structures for fucosidases from *Caenorhabditis elegans*, *Drosophila melanogaster*, and *Danio rerio* to the structure of FucA1 shows a consistent positioning of the conserved aspartate on the acid/base loop (Figure 5).

Although we have shown here that Glu289 is not the true acid/base, the structure of FucA1 does provide insight into why mutation of this residue results in a severe loss of activity. Glu289 is within hydrogen-bonding distance of both Trp223 (part of the hydrophobic pocket near the 5 position of DFJ) and Asn273, which appears to stabilize the loop where the genuine acid/base Asp276 is located (Figure S7). Replacement of Glu289 with another amino acid likely destabilizes the acid/base loop, causing the genuine acid/base to be poorly positioned for catalysis. Indeed, the Glu289Gln mutant is considerably less stable, as determined by a thermal shift assay, than the wild-type enzyme (ΔT_m_ of −12.3 ± 0.1°C; Figure S8). The Glu289Gln variant is also stabilized by DFJ (ΔT_m_ of +4.63 ± 0.09°C; Figure S8), indicating that the active site is still able to bind fucose-configured sugars. This result underscores the need for structural information to contextualize the functional role of catalytic residues.

### FucA1 structure gives insight into location of natural variants and missense mutations

**Figure 6:**
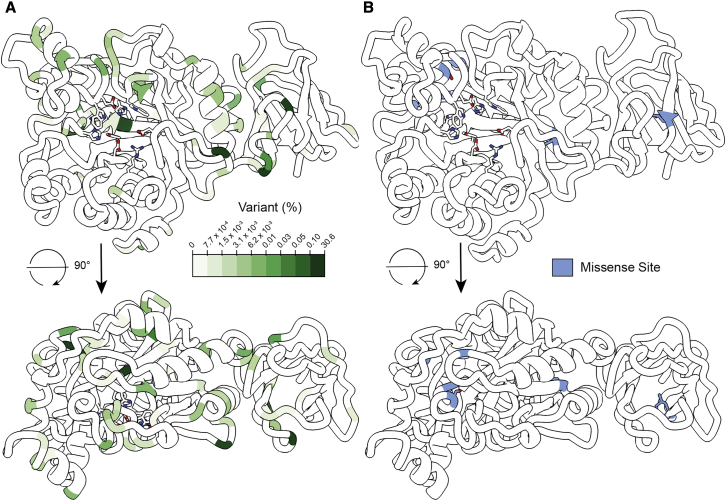
Position of natural variants and fucosidosis mutations (A) Position and abundance of natural variants of FucA1, retrieved from the genomAD database (Karczewski et al., 2020) and colored by variant population percentage. (B) Position of missense mutations found in patients with fucosidosis. Front and side views are shown for both panels.

The structure of FucA1 also gives insight into the position of both natural variants and causative mutations present in fucosidosis. To assess the position of natural variants, we inspected missense mutations from the gnomAD database (Karczewski et al., 2020) (Figure 6A). Many of the most frequent natural variants are present on external loops and α-helices; no active-site residues have any natural variation, and very few variants are present on the β-barrel core of the catalytic domain. One exception is Leu129Phe, present on strand 2 of the β-barrel, that is positioned in a hydrophobic cavity large enough to accommodate a phenylalanine (Figure S10). The most common natural variant—Gln281Arg (population frequency: 30.6%)—is present on the acid/base loop. Although this is a shift from an uncharged to cationic residue, the Gln281 side chain projects toward the bulk solvent and is not involved in binding to other regions of the protein (Figure S10).

Surprisingly, none of the missense mutations identified in cases of fucosidosis are present in the active site (Figure 6B). The six known missense mutations (see Table 3) represent either the shift from a non-polar residue to a charged residue in the protein’s interior (G60D, G340E, L405R) or a disruption of the hydrogen-bonding network of a polar residue by replacement with a non-polar/aromatic residue (S63L, S150F, N329Y). The position of these mutants suggests that all may be amenable to treatment with an appropriate pharmacological chaperone, as the catalytic machinery is not perturbed. As such, we considered whether DFJ could stabilize FucA1, analogous to migalastat treatment of Fabry disease (Fan et al., 1999). Indeed, FucA1 exhibits a considerable increase in stability (ΔT_m_ of +11.8°C; Figure S11) upon incubation with 25 μM DFJ, suggesting that DFJ-like molecules could be pharmacological chaperones.

**Table 3:** Fucosidosis missense mutations of FucA1

Mutation	Type	Reference
G60D	missense	Seo et al., 1993
S63L	missense	Seo et al., 1994
N329Y	missense	Cragg et al., 1997
S150F	missense	Fernandez-Marmiesse et al., 2014
G340E	missense	Prietsch et al., 2008
L405R	missense	Fleming et al., 1998

Encouraged by the substantial DFJ stabilization of FucA1, we next investigated whether fucosidosis-causing mutations could also be stabilized by DFJ. We attempted to express six known protein variants that have been observed in fucosidosis (Gly60Asp, Ser63Leu, Ser150Phe, Asn329Tyr, Gly340Glu, and Leu405Arg) as we did for the wild-type protein. However, western blot analysis revealed that while the Gly60Asp and Ser150Phe mutants expressed well, Ser63Leu demonstrated a clear reduction in expression levels, and Asn329Tyr, Gly340Glu, and Leu405Arg exhibited almost undetectable levels of expression (Figure S12). Reduction in levels of expression for the latter four mutants is not unexpected, as we anticipated that disease-causing variants could be misfolded and thereby targeted for protein degradation. Regardless, we attempted to express and purify all six mutants; however, only Gly60Asp and Ser150Phe provided enough material for biochemical analysis (Figure S12). During purification, the size-exclusion trace indicated that the Gly60Asp variant mostly formed high-molecular-weight species/aggregates; however, since the protein was fully soluble in solution, we decided to assay protein activity nonetheless.

To determine whether these mutants cause disease progression through impaired catalysis, instability, or a combination thereof, we assayed Gly60Asp and Ser150Phe variants against pNP-α-L-Fuc and investigated their thermal stability and tertiary structure. The catalytic activity of the Gly60Asp variant on pNP-α-L-Fuc was below the limit of detection for this assay (see Table 2). The Ser150Phe variant hydrolyzed pNP-α-L-Fuc with similar *K*_M_ values to the wild-type enzyme and exhibited a ∼13- to 20-fold reduction in k_cat_, a result that is consistent with a large subpopulation of the enzyme being misfolded. To determine the stability of Gly60Asp and Ser150Phe, we initially attempted a thermal shift assay as used for the wild-type enzyme (Figure S11); however, we observed an initial background fluorescence much greater than that of the wild-type enzyme. The fluorescent dye used in these assays has greater fluorescence when bound to hydrophobic regions of proteins, thus we reasoned that both the Gly60Asp and Ser150Phe mutations facilitated increased access to a hydrophobic section in the protein core. Due to this high fluorescence signal, we were unable to draw definitive conclusions from the data regarding protein stability; however, we did observe that pre-incubation of the mutants with DFJ did not facilitate a return to wild-type fluorescence levels in the mutants, suggesting that DFJ binding does not completely stabilize the variants. To demonstrate that these mutants are structured but differ from the wild-type enzyme, we analyzed both mutants by CD (Figure S12). Both Gly60Asp and Ser150Phe deviate in secondary structure from the wild-type enzyme.

As we were unable to determine protein melting temperatures through a dye-based thermal stability assay, we used nano differential scanning fluorimetry (nanoDSF) experiments that rely on the intrinsic fluorescence of tryptophan residues to detect unfolding (Table 4; Figure S13). We validated our methodology by incubating the wild-type enzyme again with and without DFJ and observed a shift of ΔT_m_ of +14.7 ± 0.7°C in the presence of DFJ. Both mutants were less stable than the wild-type protein (Gly60Asp, ΔT_m_ of −3.7 ± 0.2°C, and Ser150Phe, ΔT_m_ of −8.6 ± 0.2 °C). Gly60Asp was not stabilized by DFJ; this result, in combination with the kinetics and the large shift seen in the CD spectra, would appear to indicate that the active site of Gly60Asp is not configured correctly for catalysis and that DFJ may prove to be a poor pharmalogical chaperone for this variant. In contrast, Ser150Phe was stabilized by DFJ, indicating that the use of a pharmacological chaperone may be a viable therapeutic option for this variant, warranting future *in vivo* evaluation of pharmacological chaperones in a disease model.

**Table 4:** Nano-DSF of FucA1 variants

Enzyme	Ligand	T_m1_ (°C)	T_m2_ (°C)
FucA1-WT	–	54.9 ± 0.13	–
FucA1-WT	+DFJ	69.57 ± 0.52	–
FucA1-G60D	–	51.25 ± 0.1	–
FucA1-G60D	+DFJ	51.53 ± 0.3	–
FucA1-S150F	–	46.31 ± 0.11	–
FucA1-S150F	+DFJ	50.15 ± 0.71	59.57 ± 0.21

Melting temperatures determined by fluorescence emission at 330 nm.

It is difficult to understand the wide-ranging effects of fucose metabolism in humans without understanding the molecular basis of fucosidases. By determining the structure of FucA1 through single-particle cryo-EM, we have amended the annotation of the catalytic acid/base, resolving the major question surrounding this class of enzymes. Furthermore, we have shown that the catalytic acid/base is conserved in essentially all animal GH29 fucosidases. Finally, we have shown the position of fucosidosis-causing mutations and the stabilization of a specific disease-causing mutant by a small molecule, a step toward identifying molecular treatments for fucosidosis.

## STAR★Methods

### Key resources table

**Table:** 

REAGENT or RESOURCE	SOURCE	IDENTIFIER
**Antibodies**		

HRP anti-His antibody	BioLegend	# J099B12

**Bacterial and virus strains**		

*E. coli* DH10emBacY	Geneva Biotech	# EMBacY
*E. coli* DH5α cells	NEB	# C2987H

**Chemicals, peptides, and recombinant proteins**		

Deoxyfuconojirimycin	Dextra Laboratories	IS0785
FuGENE HD transfection reagent	Promega	E2311
pNP α-L-fucopyranoside	MilliporeSigma	10231-84-2
fucose-α-1,2-galactose	Carbosynth	24656-24-4
SYPRO™ orange protein dye	ThermoFisher	S6650

**Critical commercial assays**		

Fucose detection assay	Megazyme	K-FUCOSE

**Deposited data**		

Cryo-EM structure of unliganded FucA1	This work	EMDB-13499, PDB 7PLS
Cryo-EM structure of FucA1-Deoxyfuconojirimycin complex	This work	EMDB-13520 PDB 7PM4

**Experimental models: Cell lines**		

SF9 cells (*Spodoptera frugiperda)*	Invitrogen	11496015
High Five cells (*Trichoplusia ni*)	Invitrogen	B85502

**Oligonucleotides**		

CTATAACTGTGAAAATAAATTCAAGCCAC	IDT	D276N_F
GTGGCTTGAATTTATTTTCACAGTTATAG	IDT	D276N_R
CAGATCACAAGTGGCAGATGTGCACCAG	IDT	E289Q_F
CTGGTGCACATCTGCCACTTGTGATCTG	IDT	E289Q_R
GTTCATCCACTGGGACGTGTTCTCGGTG	IDT	G60D_F
CACCGAGAACACGTCCCAGTGGATGAAC	IDT	G60D_R
GGGGCGTGTTCTTGGTGCCCGCCTG	IDT	S63L_F
CAGGCGGGCACCAAGAACACGCCCC	IDT	S63L_R
CTTGGAACTGGAACTTCAAAGACGTGGGGC	IDT	S150F_F
GCCCCACGTCTTTGAAGTTCCAGTTCCAAG	IDT	S150F_R
CAGTAAGTTTGGGAGGCTACTATCTTCTGAACATTG	IDT	N329Y F
CAATGTTCAGAAGATAGTAGCCTCCCAAACTTACTG	IDT	N329Y R
GGACCAACTAAAGATGAACTGATTGTTCCCATC	IDT	G340E F
GATGGGAACAATCAGTTCATCTTTAGTTGGTCC	IDT	G340E R
GAAAATGGAGTCTTAAACCGTGAATCCCCCATAACTAC	IDT	L405R F
GTAGTTATGGGGGATTCACGGTTTAAGACTCCATTTTC	IDT	L405R R

**Recombinant DNA**		

pOmniBac1_FucA1	This Work	N/A
pOmniBac1_FucA1_D276N	This Work	N/A
pOmniBac1_FucA1_E289Q	This Work	N/A
pOmniBac1_FucA1_G60D	This Work	N/A
pOmniBac1_FucA1_S63L	This Work	N/A
pOmniBac1_FucA1_S150F	This Work	N/A
pOmniBac1_FucA1_N329Y	This Work	N/A
pOmniBac1_FucA1_S63L	This Work	N/A
pOmniBac1_FucA1_G340E	This Work	N/A
pOmniBac1_FucA1_L405R	This Work	N/A

**Software and algorithms**		

Astra V software	Wyatt technology	https://www.wyatt.com/products/software/astra.html
ChimeraX 1.2.5	(Pettersen et al., 2020)	https://www.cgl.ucsf.edu/chimerax/
Coot 0.8.9.2	(Emsley et al., 2010)	https://www2.mrc-lmb.cam.ac.uk/Personal/pemsley/coot/
GCTF	(Zhang, 2016)	https://www2.mrc-lmb.cam.ac.uk/research/locally-developed-software/
GraphPad Prism 5.0	GraphPad Software	https://www.graphpad.com/
MolProbity	(Williams et al., 2018)	http://molprobity.biochem.duke.edu/
Motioncorr2	(Zheng et al., 2017)	https://msg.ucsf.edu/
Phenix 1.19	(Liebschner et al., 2019)	https://www.phenix-online.org
Pymol 2.3.0	Schrodinger LLC	https://www.pymol.org/2/
Relion-3	(Zivanov et al., 2018)	https://www3.mrc-lmb.cam.ac.uk/relion/

### Resource availability

#### Lead contact

Further information and requests for resources and reagents should be directed to and will be fulfilled by Gideon J. Davies (gideon.davies@york.ac.uk).

#### Materials availability

Plasmids generated in this study are available upon request.

### Experimental model and subject details

#### Cell lines

High Five, and Sf9 cells were purchased from Invitrogen. The cells were used directly from the commercial sources following manufacturer suggestions as described in detail below.

### Method details

#### Production of FucA1-expressing baculovirus

A codon optimized version of FucA1 was synthesized by Genscript (Leiden, the Netherlands). DNA encoding for amino acids 32–466 of FucA1 was subcloned into the pOMNIBac vector (Geneva Biotech), behind a honeybee melittin secretion peptide, hexahistidine tag, and TEV cleavage site. Recombinant bacmid was produced with the Tn7 transposition method in DH10EMBacY (Geneva Biotech)(Bieniossek et al., 2012) and purified with the PureLink miniprep kit (Invitrogen) according to standard protocols. V1 baculovirus was produced by transfection of bacmid into low-passage adherent Sf9 cells (Invitrogen) with FuGENE HD transfection reagent (Promega), at a ratio of 2 μg DNA to 4.5 μL FuGENE. V1 to V2 virus amplification was carried out with Sf9 cells in suspension culture, and the YFP marker present in EMBacY baculovirus was used to determine optimum amplification before harvesting (typically ∼60% cells fluorescent). For expression, High Five™ (*Trichoplusia ni*) cells (Invitrogen) were infected with V2 baculovirus at an MOI >1, and infection followed with the EMBacY YFP marker to determine the optimum time point for harvesting (typically 72 h, with >80% cells fluorescent).

#### Expression and purification of wild-type FucA1 and the and D276N variant

Conditioned medium (3.6 L) was cleared of cells by centrifugation at 500 x *g* for 15 min at 4°C; this was followed by further clearing of debris by centrifugation at 5,000 x *g* for 30 min at 4°C. DTT (1 mM) and PMSF (0.1 mM) were added to clarified medium, which was then loaded onto a pre-equilibrated HisTrap excel 5-mL column (GE Healthcare). The HisTrap column was washed with 10 CV buffer A (20 mM HEPES, 100 mM NaCl, 20 mM Imidazole pH 7.4, 1 mM DTT) and eluted with a linear gradient over 20 CV with buffer B (20 mM HEPES, 100 mM NaCl, 500 mM Imidazole pH 7.4, 1 mM DTT). FucA1-containing fractions were pooled, diluted ten-fold into IEX buffer A (20 mM NaCl, 20 mM HEPES, 1 mM DTT, pH 7.4), and loaded onto a pre-equilibrated HiTrap Q FF anion exchange column (GE Healthcare). The HiTrap Q column was then washed with 5 column volumes of IEX buffer A, then FucA1 eluted from the HiTrap Q column with a linear gradient over 20 CV with IEX buffer B (1.5 M NaCl, 20 mM HEPES, 1 mM DTT, pH 7.4). FucA1-containing fractions were pooled and concentrated to a volume of ∼1 mL with a 30-kDa Vivaspin concentrator (GE Healthcare) and treated with AcTEV protease (Thermo Fisher) at ambient temperature overnight to remove the N-terminal His-tag. Digested protein was passed through a HisTrap excel 5-mL column (GE Healthcare) that had been pre-equilibrated with buffer A and the flowthrough was collected. This flowthrough was concentrated as above and further purified by size-exclusion chromatography (SEC) with a Superdex S200 16/600 column (GE Healthcare) in SEC buffer (20 mM HEPES, 200 mM NaCl, 1 mM DTT, pH 7.4). FucA1-containing fractions were concentrated to 2 mg/mL with a 30-kDa Vivaspin concentrator. Small aliquots were flash frozen in liquid nitrogen and stored at −80°C until use.

#### Mutagenesis

Mutagenesis was performed using a modified QuikChange™ (Agilent) protocol. For each mutant generated, PCR was first performed for 12 cycles with one of the sense or antisense primers (D276N_F; D276N_R); these two reactions were subsequently pooled, and an additional 18 cycles of PCR were performed. PCR products were digested with DpnI (New England Biolabs) and transformed into chemically competent DH5α cells. Purified plasmids were sequenced to confirm the correct mutation prior to baculovirus production. The acid/base variant was expressed and purified as for the WT protein.

#### Western blot analysis of disease mutants

Flasks containing 50 mL of High Five™ cells (*Trichoplusia ni*) at a density of 1.9 × 10^6^ were infected with 83 μL of V2 and incubated at 28°C in a shaking incubator until between 60-80% of cells displayed the YFP marker (∼72 hours, all mutants displayed similar numbers of fluorescent cells and were harvested at the same time). Conditioned media was clarified by centrifugation at 50 x *g* for 20 min at 4°C before a further centrifugation step at 3894 x *g* for 20 mins at 4°C. Conditioned media was mixed 1:1 with 2 x SDS-PAGE sample loading buffer. Samples were resolved on a 10% SDS-PAGE gel, the volume of each sample loaded was normalised to the total number of fluorescent cells counted before harvesting. Standard western blot procedures were followed using a HRP anti-His antibody (BioLegend, #J099B12, 1:5000). Western blot was developed with a Western-Ready™ ECL substrate detection kit (Biolegend) and imaged on an Invitrogen iBright FL1000 imaging system.

#### Expression and purification of E289Q and disease mutants

These mutants were produced on a smaller scale and the protocol deviates slightly from wild-type protein owing to this. Conditioned media (600 mL) was clarified and purified by His-tag affinity identically to the wild-type protein. Protein fractions containing FucA1 were pooled and dialysed against wild-type SEC buffer with in-house TEV protease overnight at ambient temperature. Dialysed samples were passed through a HisTrap excel 5-mL column (GE Healthcare) equilibrated in buffer A to remove the N-terminal His-tag and His-tagged TEV protease. Flowthroughs were collected and concentrated using 30-kDa Vivaspin concentrators before samples were further purified by SEC using a Superdex 200 Increase 10/300 GL column in SEC buffer. FucA1-containing fractions were concentrated as before and flash frozen in liquid nitrogen. Samples were stored at −80°C before use. Purity of samples was assessed by SDS-PAGE (Extended Data Figures S8 and S12).

#### Cryo-EM data collection

A frozen aliquot of FucA1 at 2 mg/mL was thawed rapidly, then kept on ice until use. A total of 2.5 μL of this sample was applied to glow-discharged R1.2/1.3 300 mesh UltrAuFoil gold grids (Quantifoil), which were subsequently blotted for 2 s with a blot force of −10 before being plunged into liquid ethane cooled by liquid nitrogen. Plunge-freezing was performed using a Vitrobot Mark IV (Thermo Fisher Scientific) at 100% humidity and 22°C.

For enzyme-inhibitor complexes, phosphate citrate buffer, pH 4.5 was added to the frozen enzyme stocks to a final concentration of 60 mM. Deoxyfuconojirimycin was added to achieve an inhibitor concentration of 1 mM and a FucA1 concentration of 1.5 mg/mL. This mixture was then incubated at 37°C for 30 min prior to plunge-freezing as above.

EER formatted movies of FucA1 were acquired on the Glacios microscope (Thermo Fisher Scientific), housed in the York Structural Biology Laboratory. The microscope was operated at an accelerating voltage of 200 kV with a Falcon 4 direct electron detector. The unliganded FucA1 Dataset was acquired at a dose rate of 6.4 electrons per pixel per second, and a pixel size of 0.934 Å; target defocus values were −1.2, −1.5 and −1.8 μm. The FucA1-DFJ data set was collected at a dose rate of 14 electrons per pixel per second, and a pixel size of 0.574 Å; target defocus values were −1.2, −1.5 and −1.8 μm. The autofocus function was run every 10 μm. Both datasets were collected with a total dose of 50 electrons per Å^2^.

#### Image processing and 3D reconstruction

Movie frames of the uncomplexed FucA1 were motion corrected without binning, using a pixel size of 0.934 Å, and dose-weighted using the Motioncorr2 program(Zheng et al., 2017). CTF corrections were performed using the GCTF program(Zhang, 2016). Most of the subsequent processing steps were carried out using RELION 3(Scheres, 2012). LoG based automated particle picking was performed on the data. Particles were extracted and subjected to 2D classification. 2D classes showing sharp structural features were chosen to build an initial 3D model. This initial model was then used for 3D classification, without the use of symmetry constraints. The class showing well defined structural features was then selected for 3D refinement with D2 symmetry imposed. This gave a reconstruction with a resolution of 3.1 Å. Subsequently, CTF refinement(Zivanov et al., 2018) was performed for magnification anisotropy; optical aberrations (up to the fourth order); and per-particle defocus and per-micrograph astigmatism. This was followed by Bayesian polishing(Zivanov et al., 2019) to optimise per-particle beam-induced motion tracks, followed by another round of auto-refinement. CTF refinement was then repeated for optical aberration correction, magnification anisotropy, per-particle defocus and per-micrograph astigmatism, yielding a 2.49 Å map.

The unliganded structure was used as a model for particle picking and an initial model for 3D refinement for liganded structures. The selected particles were processed as for the unliganded structure. Further details of the image processing and 3D reconstruction can be found in Table 1.

#### Model building, refinement, and validation

An initial model was built using the map_to_model function in Phenix (Liebschner et al., 2019). This initial model was modified using COOT (Emsley et al., 2010). Real-space refinement was carried out with secondary structure restraints using Phenix (Liebschner et al., 2019). Model geometries were assessed with MolProbity (Williams et al., 2018). Structures and maps in the figures were rendered with PyMOL (http://www.pymol.org/) or ChimeraX (Pettersen et al., 2020).

#### SEC-MALLS

Experiments were conducted on a system comprising a Wyatt HELEOS-II multiangle light scattering detector and a Wyatt rEX refractive index detector, linked to a Shimadzu HPLC system (SPD-20A UV detector, LC20-AD isocratic pump system, DGU-20A3 degasser and SIL-20A autosampler). Work was conducted at room temperature (20 ± 2°C). Sample injection volume was 100 μL at a protein concentration of 0.8 mg/mL. The samples were separated on a Superdex S200 10/300 (GE Healthcare) using 20 mM HEPES (pH 7.4), 200 mM NaCl as buffer. Shimadzu LC Solutions software was used to control the HPLC and Astra V software for the HELEOS-II and rEX detectors. Data were analyzed using the Astra V software. Molecular weights were estimated using the Zimm fit method (Zimm, 1945) with 1 degree. A value of 0.182 was used for protein refractive index increment (dn/dc).

#### Activity assays

All activity assays were performed at 37°C in a buffer containing 32 mM sodium phosphate, 15 mM sodium citrate (pH 5.0), and 100 mM NaCl, and were initiated by the addition of the appropriate amount of enzyme. Kinetic parameters were determined for pNP α-L-fucopyranoside using substrate concentrations between 50 μM and 2 mM. Assays were performed in 96-well-plates and each plate included pNP standards in an identical buffer system for concentration calibration. pNP α-L-Fuc hydrolysis was measured after stopping the assay with a 1:1 ratio of 1 M Na_2_CO_3_ (pH 11.2). Absorbance at 405 nm was measured with a CLARIOstar Plus plate reader (BMG Labtech). Kinetic parameters for the hydrolysis of fucose-α-1,2-galactose were measured using the L-fucose assay kit (Megazyme; K-FUCOSE) according to the manufacturers recommended procedures for microplate use.

#### Thermal-shift assays with SYPRO™ orange protein dye

A reaction (50 μL total) of wild-type FucA1 (2.5 μM) with 5 x SYPRO™ orange protein dye with or without 25 μM DFJ, in 50 mM McIlvaine buffer pH 5.0 and 200 mM NaCl, was incrementally raised to 94.5°C from 24.3°C using a Stratagene Mx3005P qPCR system. An excitation of wavelength of 517 nm and emission of 585 nm were used to detect fluorescence. Three technical repeats were performed for both the unliganded and DFJ complexed samples. Each repeat was normalised to their maximum fluorescence value then fit to a Boltzmann model. GraphPad Prism 5 was used for analysis. Mutant proteins and wild-type controls were analysed identically, except 25 x SYPRO™ was used, in an attempt to maximise signal in samples with the high initial fluorescence.

#### Circular dichroism

Proteins were buffer exchanged into 50 mM potassium phosphate buffer pH 7.5 using 0.5 mL Zeba™ Spin Desalting Columns, 7K MWCO by manufacturer’s instructions. Proteins were diluted in this same buffer to a concentration of 0.15 mg/mL protein. Spectra were collected in a 0.1 cm pathlength quartz cuvette on a JASCO J-1500 instrument from 190-260 nm. Spectra were baseline corrected by subtracting a buffer only sample from the raw data. Data was plotted using GraphPad Prism 5.

#### Differential scanning fluorimetry using intrinsic protein fluorescence

Protein samples in 50 mM potassium phosphate buffer pH 7.5 (see circular dichroism) at 3 μM were analysed in the presence or absence of 15 μM DFJ by nanoDSF through a Prometheus NT.48 instrument (NanoTemper Technologies GmbH). Samples were loaded into standard Prometheus NT.48 capillaries (NanoTemper Technologies GmbH). Temperature was increased by 1°C/min from 20 to 100°C and tryptophan fluorescence was detected at 330 nm. A minimum of four technical repeats for each sample were performed. Data was normalised to an arbitrary initial fluorescence of 1 and plotted using GraphPad Prism 5.

### Quantification and statistical analysis

Cryo-EM data were processed and analyzed using Relion-3 (Zivanov et al., 2018). Cryo-EM structural statistics were analyzed with Phenix (Liebschner et al., 2019) and Molprobity (Williams et al., 2018). Statistical details of experiments are described in method details or figure legends.

## Data Availability

Cryo-EM maps have been deposited to the EMDB with accession codes: EMDB-13499 and EMDB-13520. Coordinates fit to the maps have been deposited in the PDB with accession codes: 7PM4 and 7PLS. This paper does not report original code. Any additional information required to reanalyze the data reported in this paper is available from the lead contact upon request.

## Supplemental information

Document S1. Figures S1–S13

Document S2. Article plus supplemental information
