# Supplementary material for: Cryo-EM structures of human fucosidase FucA1 reveal insight into substrate recognition and catalysis
Source: Structure. 2022 Oct 6;30(10):1443–1451.e5. doi: 10.1016/j.str.2022.07.001 (PMC9548408; doi:10.1016/j.str.2022.07.001)
Supplement: Document S1. Figures S1–S13 [file mmc1.pdf]

Structure, Volume *30*

Supplemental Information

Cryo-EM structures of human fucosidase FucA1 reveal insight into substrate recognition and catalysis

Zachary Armstrong, Richard W. Meek, Liang Wu, James N. Blaza, and Gideon J. Davies

Cryo-EM structures of human fucosidase FucA1 reveal insight into substate recognition and catalysis.

Authors : Zachary Armstrong, Richard W. Meek, Liang Wu, James N. Blaza, Gideon J. Davies

Supplemental Figures

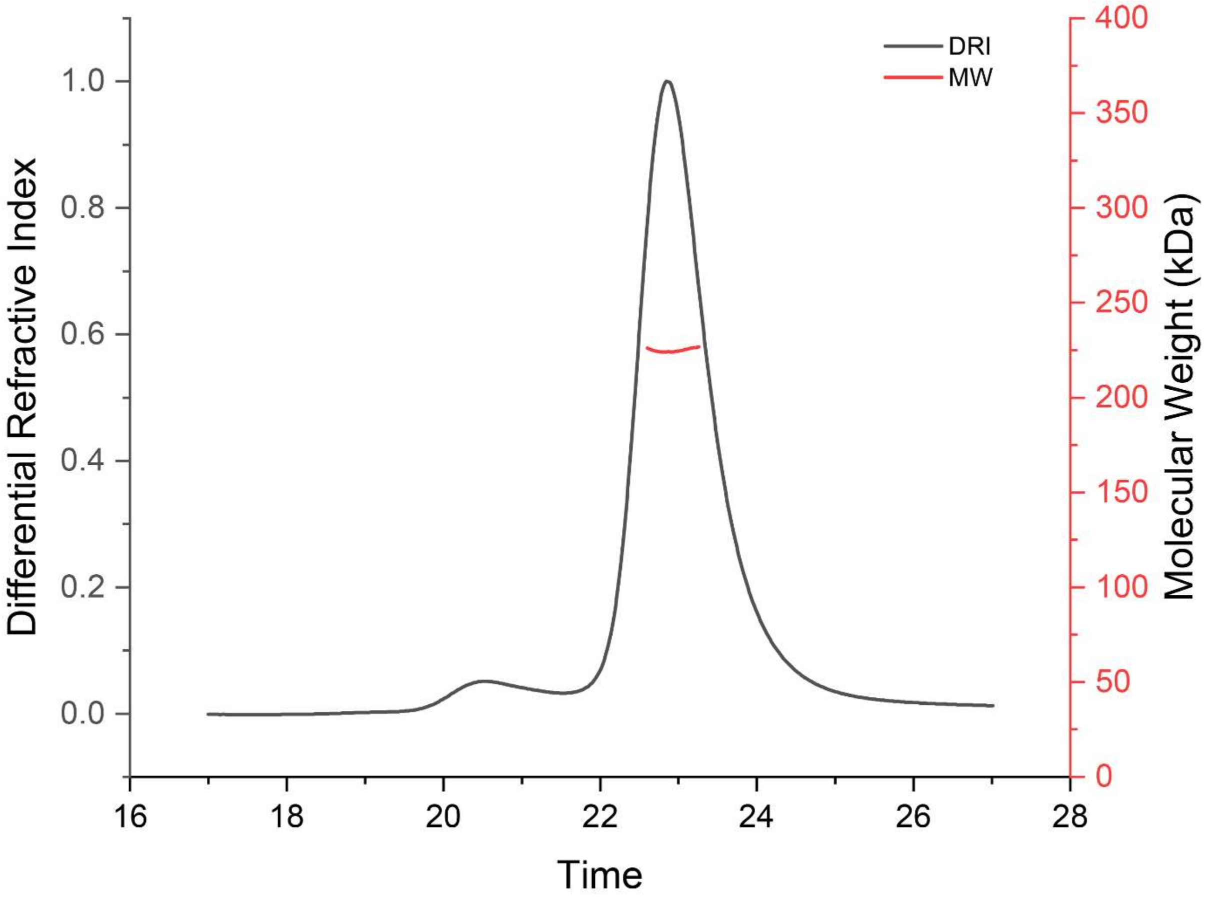

Figure S1. SEC-MALLS trace of purified FucA1. Related to Figure 1. The average molecular weight of proteins eluting in the largest peak was determined to be 225 kDa.

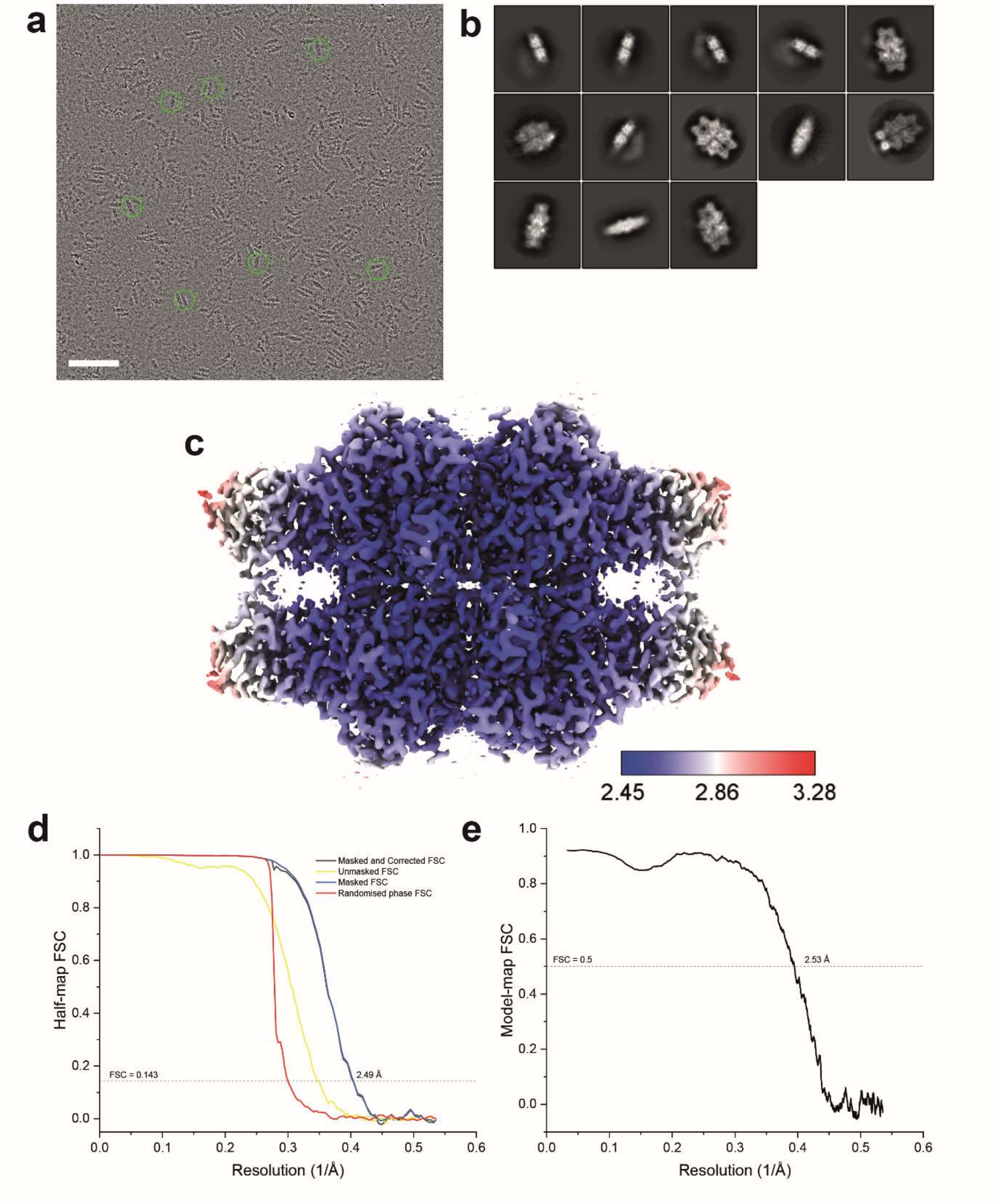

Figure S2. Cryo-EM data processing for unliganded FucA1. Related to Figure 1. **(a)** Representative micrograph of FucA1 from a hole on a UltrAuFoil R1.2/1.3 300 mesh gold grid that had been glow-discharged for 3 mins at 20 mA/0.38 mBar. Green circles indicate a few picked particles from the micrograph. White scale bar is 500 Å in length. **(b)** 2D class averages generated from auto-picked particles prior to 3D classification. **(c)** Local resolution map (**d**) Fourier Shell Correlation (FSC) between the two independently refined half-maps. **(e)**, FSC between the model and the map calculated for the model refined against the full reconstruction (black). Atomic models were refined including spatial frequencies up to 2.45 Å.

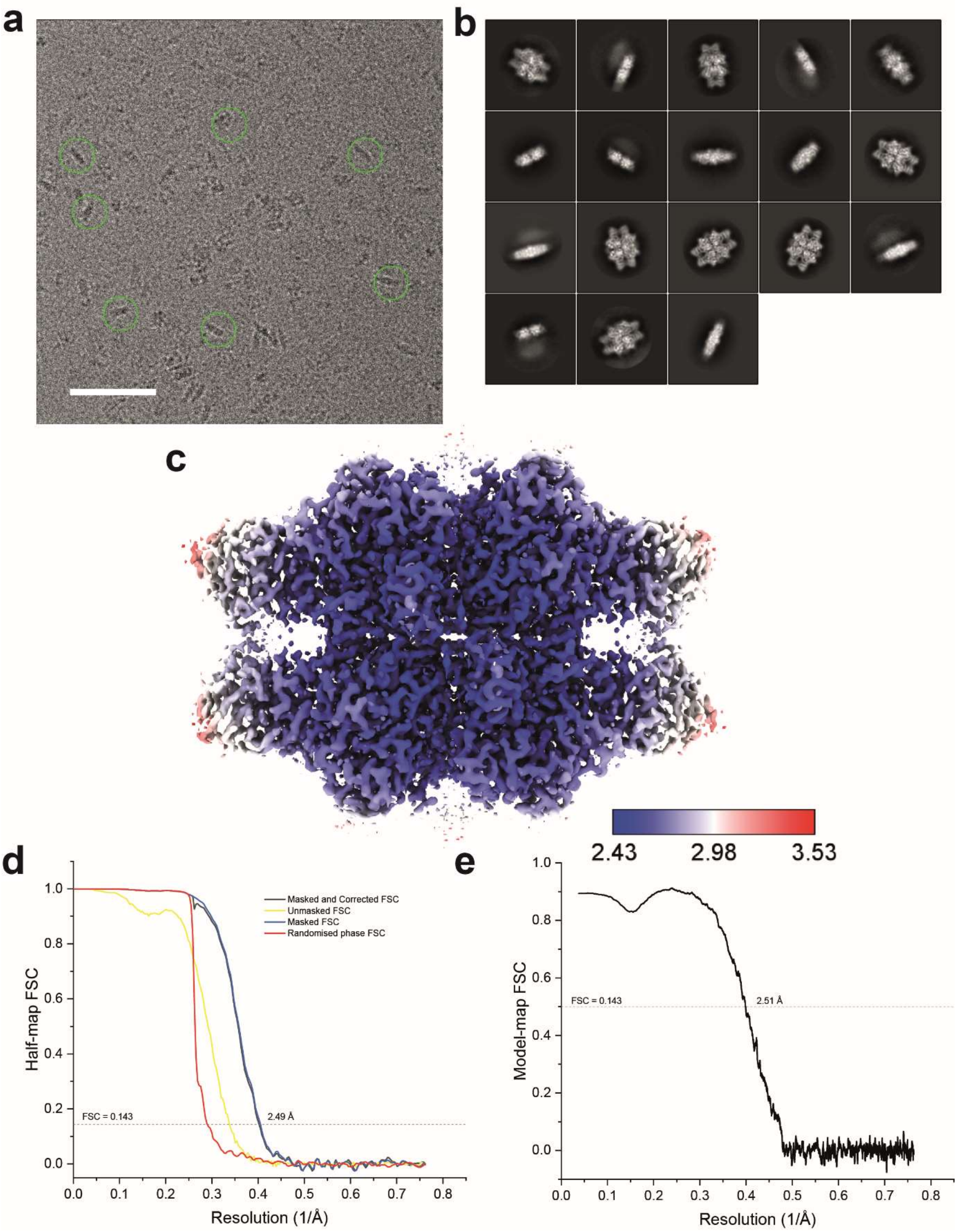

Figure S3. Cryo-EM data processing for DFJ-FucA1. Related to Figure 3. **(a)** Representative micrograph of DFJ-FucA1 from a hole on a UltrAuFoil R1.2/1.3 300 mesh gold grid that had been glow-discharged for 3 mins at 20 mA/0.38 mBar. Green circles indicate a few picked particles from the micrograph. White scale bar is 500 Å in length. **(b)** 2D class averages generated from auto-picked particles prior to 3D classification. **(c)** Local resolution map (**d**) Fourier Shell Correlation (FSC) between the two independently refined half-maps. **(e)**, FSC between the model and the map calculated for the model refined against the full reconstruction (black). Atomic models were refined including spatial frequencies up to 2.45 Å.

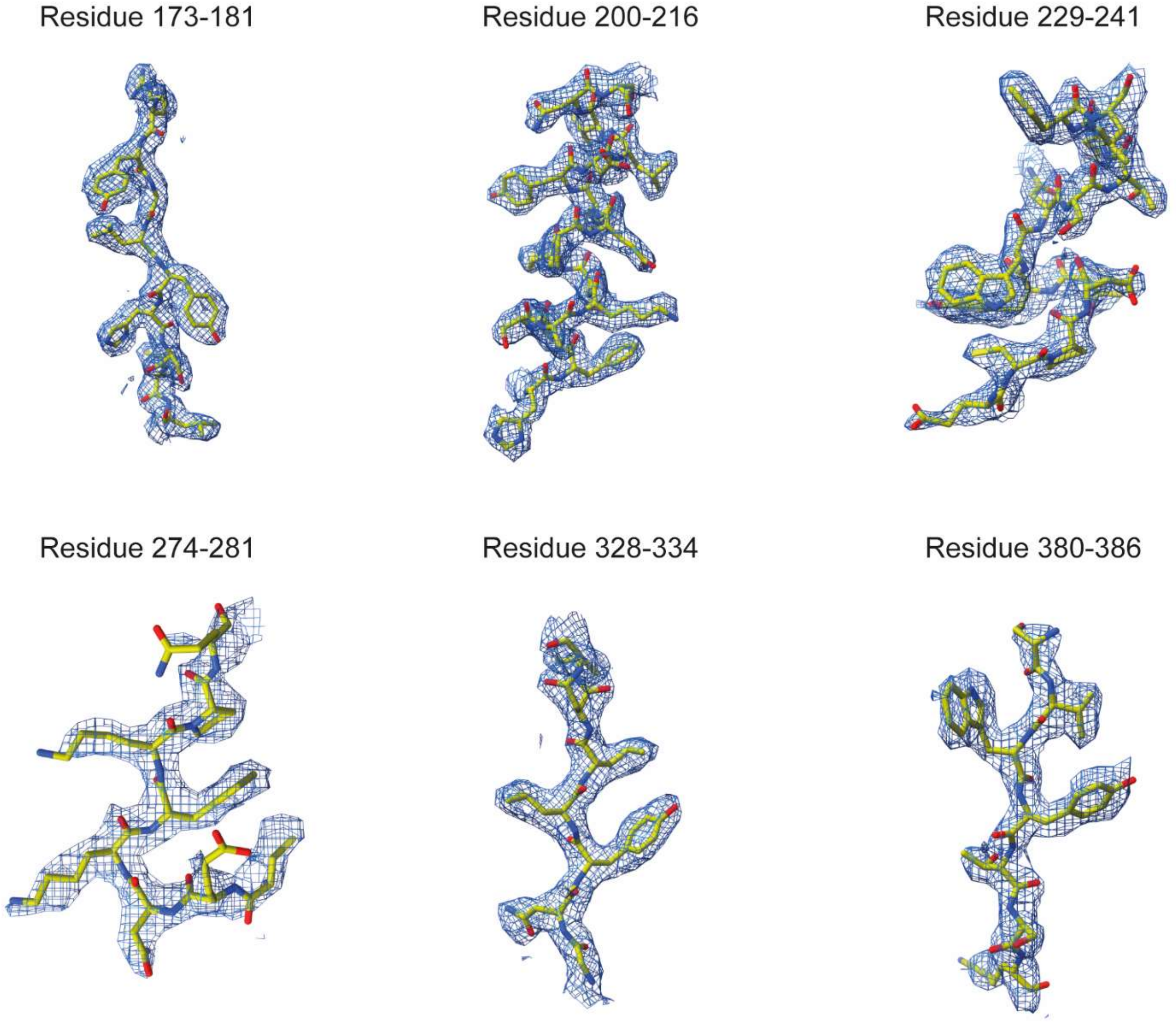

Figure S4. Representative densities of FucA1. Related to Figure 1. Representative densities taken from different parts of the unliganded FucA1 map using a threshold of 0.03-0.04.

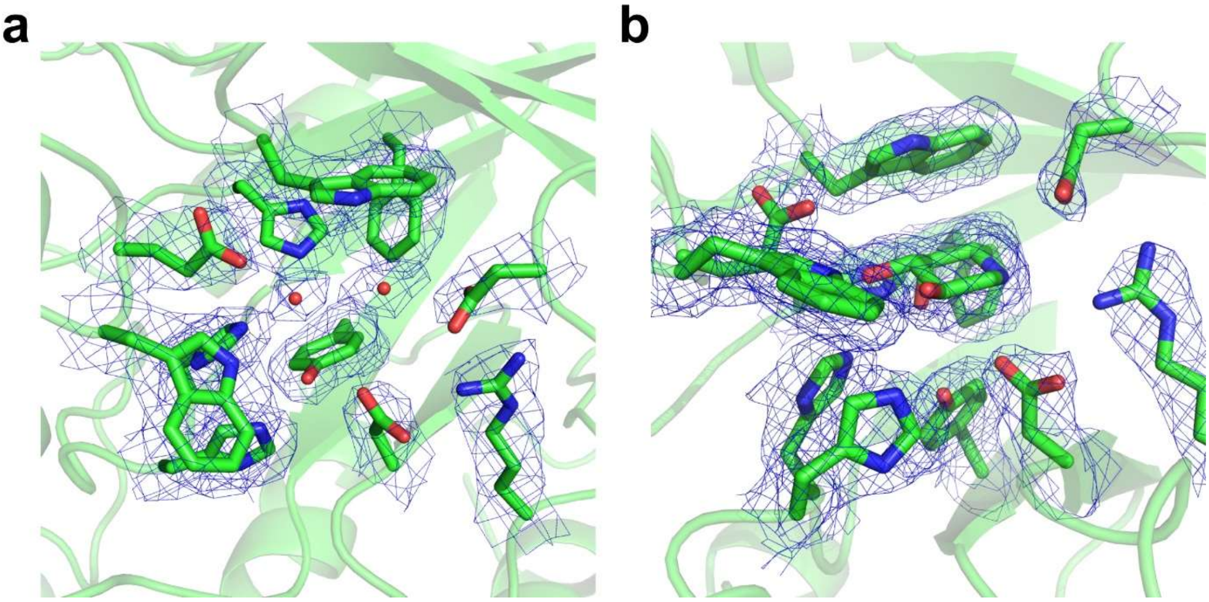

Figure S5. Active site Density Maps. Related to Figure 3. **a)** Active site density map of unliganded FucA1 and **b)** FucA1 complexed with deoxyfuconojirimycin. Potential maps are shown at thresholds of 0.028 and 0.016 for the unliganded and FucA1-DFJ maps respectively.

```
                                         *N                                                  *A
Class A  Hs FucA1      223  K---P-DLIWSDGEWECP--D-TYW--NSTNFL-SWLYNDSP[3]EVVVND     RWGQNCSCHH-------G--    --
         Hs FucA2      221  Q---P-EVLWSDGDGGAP--D-QYW--NSTGFL-AWLYNESP[3]TVVTND     RWGAGSICKH-------G--    --
         T. maritima   217  L---P-DVLWNDMGW--P--E-KGK--EDLKYLFAYYYNKHP[3]---VND     RWG----VPH-------W--    --
         P. thiamin.   178  G---TIDVLWFDFSYEDM--TGEKW--KATELV-KMIRELQP   NVLIDN     RLGGNIKARE-------P--    --
Class B  B. theta.     180  G---KIWETWWDGAGADELT--TPV--YR--HWYKIVREKQP   DCVIFG[10]RWMGNEAGEA--GDPCWA--    TT
         R. gnavus     232  GndgHFVEVWMDGAKGSGAN--AQE--YDFKKWFKTIQDNEG[7]DCMLFG[ 7]RWIGNELGIA--GKDTWSKS   KV
         S. pneumoniae 160  GnagKFAEVWMDGARGEGAQ--KVN--YEFEKWFETIRDLQG   DCLIFS[ 6]RWIGNERGYA--GDPLWQKV   NP
         B. ovatus     196  G---EVHEVWFDGANGEGPNGKKQV--YDWDAFYQTIQRLQP   KAVM--[ 7]RWVGNEKGVG--RETEWN--[4]TP
         B. longum     164  G---PIFSVWLDGANGEGKNGKTQY--YDWDRYYNVIRSLQP   DAVI--[ 7]RWAGNEAGHV--RDNEWSVV[9]TM
                                  *A
Class A  Hs FucA1      275  GYYNCEDKFK-PQSLPDH   ----------KW---EMCTSIDkFSWGYRRDMAlSDVTEESEIISELVQTVSLGGNYLL
         Hs FucA2      273  GFYTCSDRYN-PGHLLPH   ----------KW---ENCMTIDkLSWGYRREAGiSDYLTIEELVKQLVETVSCGGNLLM
         T. maritima   261  DFKTAEYHVNYPGDLPGY   ----------KW---EFTRGIG-LSFGYNRNEGpEHMLSVEQLVYTLVDVVSKGGNLLL
         P. thiamin.   229  EIYAG-DFASPEQLLPPH   GivnEDGKPLPW---EACITLN-HHWGYHAHDR--DYKTPKQVVRGLVECVSKNGNMLL
Class B  B. theta.     247  DSVAIRDEAQYYKGLNE-   ----GMLDGDAYipaETDVSIR-PSWFYHAEED-SRVKSVRELWDIYCTSVGRNSVLLL
         R. gnavus     310  DKDKNTINSNKQ------[1]NatvGFEDGDQWtvpEADARIT-SGWFWGTKKN-TP-KTMEELSDMYFNSVGHNATLLL
         S. pneumoniae 230  DKLGTEAELNYL------[1]H---GDPSGTIFsigEADVSIR-PGWFY-HEDQ-DP-KSLEELVEIYFHSVGRGTPLLL
         B. ovatus     266  GIYARSQENNKRLGVFSK[7]K-ilEKATELFWypsEVDVSIR-PGWFYHAEED-GKVKSLKHLSDIYFQSVGYNSVLLL
         B. longum     241  EKSQQEDDASFATTVSSQ[7]EavaGYGDNVCWypaEVDTSIR-PGWFYHQSED-DKVMSADQLFDLWLSAVGGNSSLLL
```

Figure S6. Abbreviated multiple sequence alignment of selected GH29 α-L-fucosidases. Related to Figure 3. Sequences of both human fucosidases FucA1 (GenbankID: AAA52481.1) and FucA2 (GenbankID: CAB53746.1) were aligned with the structurally characterized fucosidases from *Thermotoga maritima* (GenbankID: AAD35394.1), *Paenibacillus thiaminolyticus* (GenbankID: CBM40947.1), *Bacteroides thetaiotaomicron* (GenbankID: AAO78076.1), *Ruminococcus gnavus* (GenbankID: PLT74914.1), *Streptococcus pneumoniae* (GenbankID: AAK76203.1), *Bacteroides ovatus* (GenbankID: ALJ46339.1) and *Bifidobacterium longum* (GenbankID: ACJ53394.1). Sequences were aligned using COBALT. The nucleophile residues are labelled above with an ***N** and highlighted with grey while the acid/base residues are labelled with ***A** and highlighted with grey.

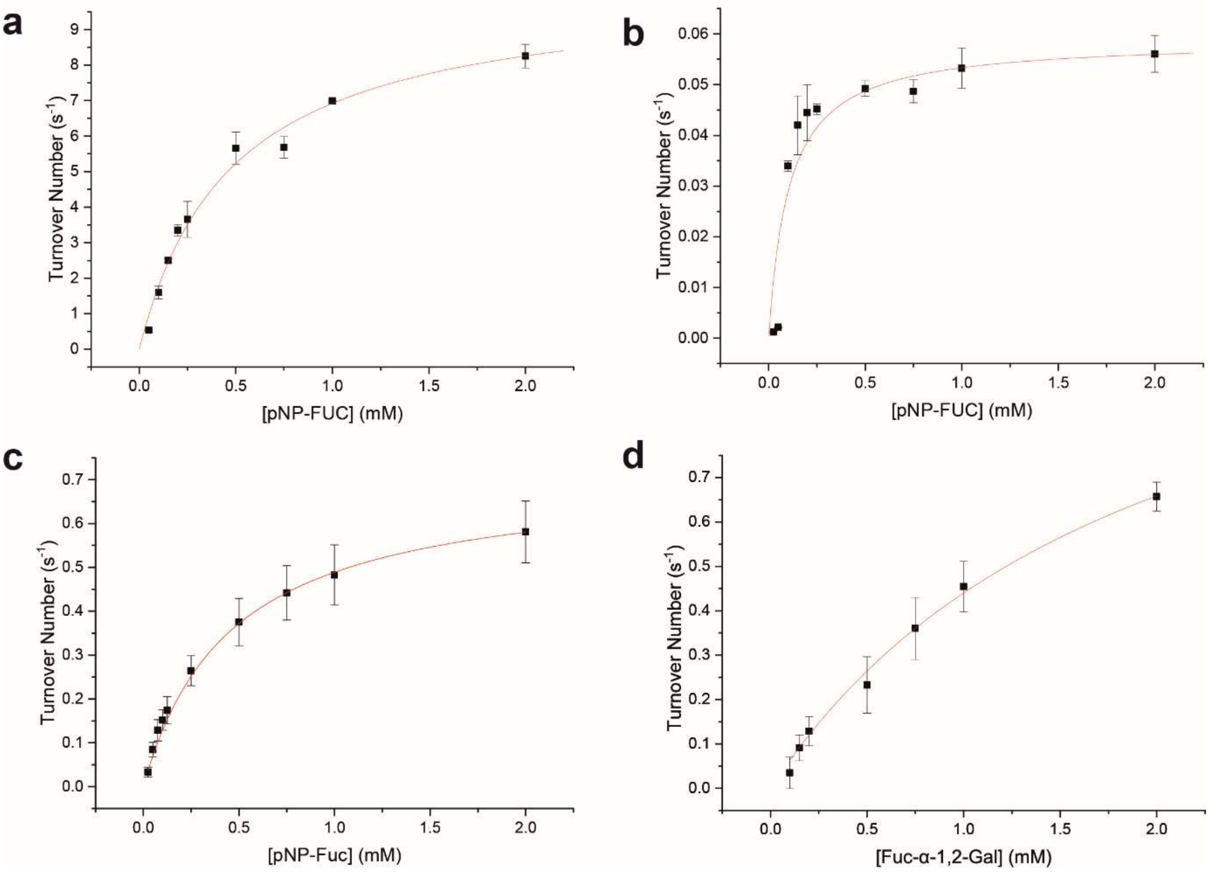

Figure S7. Michaelis-Menten plots of FucA1 activity. Related to Table 2. **a)** Wild-type FucA1 activity on pNP-FUC, **b)** FucA1 D276N variant activity on pNP-FUC, **c)** FucA1 S150F variant activity on pNP-FUC, **d**) Wild-type FucA1 activity on fucose-α-1,2-galactose. Error bars represent standard error from triplicate measurements.

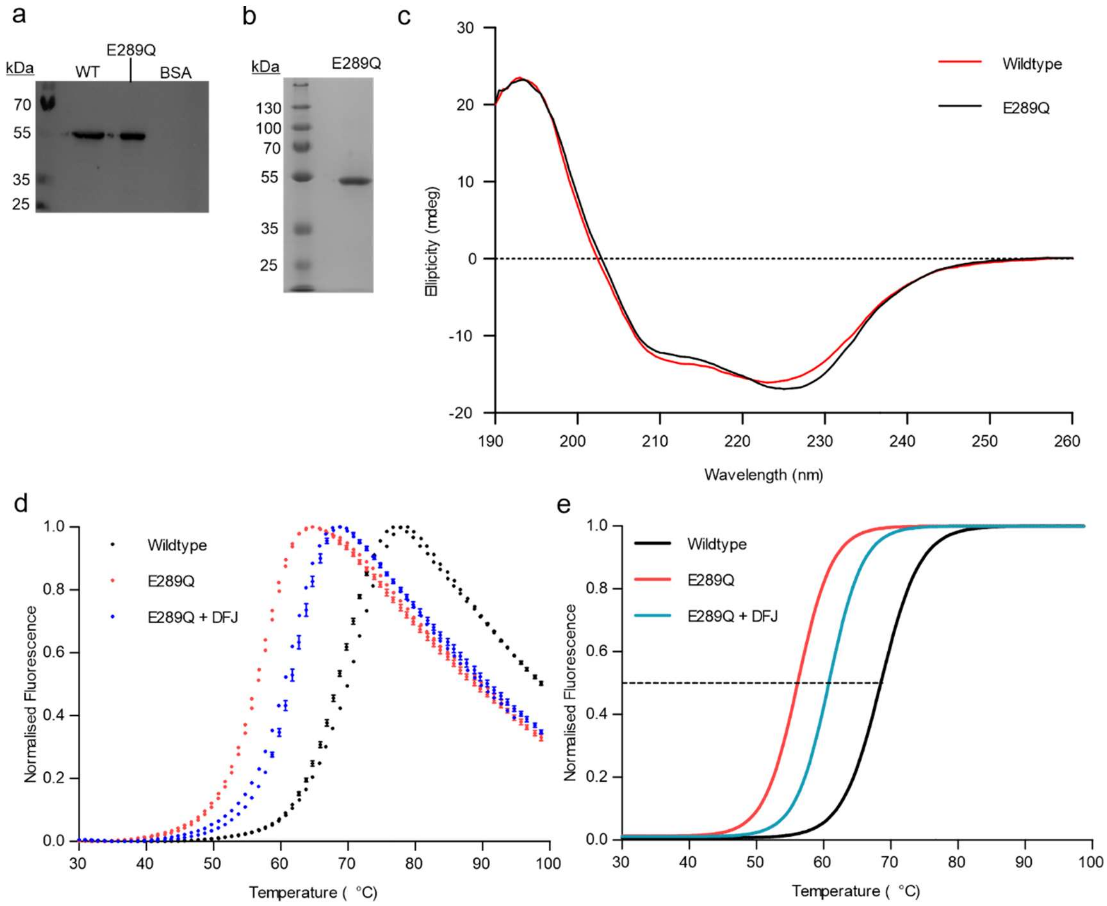

Figure S8. Characterising the Glu289Gln FucA1 variant. Related to Table 2. **a)** Western blot from expression trials, 100 ng of BSA loaded as negative control. No clear difference in levels of expression were observed between Glu289Gln and wild-type FucA1. **b)** SDS-PAGE gel of purified Glu289Gln. **c)** Circular dichroism spectra of Glu289Gln against wild-type FucA1. **d)** Thermal shift analysis of Glu289Gln with and without 25 µM DFJ compared to wild-type FucA1. Values normalised to maximum fluorescence level ± standard deviation. **e)** Fit of thermal shift data to a Boltzmann model. Calculated T_m values are 68.55 ± 0.06 °C for wild-type FucA1, 56.19 ± 0.04 °C for Glu289Gln, and 60.82 ± 0.05 for Glu289Gln in the presence of DFJ.

		*
Lepeophtheirus salmonis CAB4054172	227	GSFYSGP**D**RYNPGHILKHKFEEALTLDKKSWGFRHEISCADVLSASELVQ
Ceratitis capitata ACI01844	267	GDVYNCA**D**RFNPGVLQAHKWENAFTLDRTTWGPRFDVSFADLMTSKEVIK
Bombyx mori BCX80342	270	GDFYNHQ**D**RFNPGVLLHHKWENAFTVDSGSWGYRRNMQIREILTIEELLQ
Dendroctonus ponderosae AEE62189	255	GDIHTCE**D**RYNPGYLLDHKWENAMTIDWNSWGYRRNSNIGEYATTKQLID
Schistosoma japonicum AAP05896	273	GGYFSCD**D**HYRPGKLVRHKWENCMTLDCCSWGFRREISLDKILTPEQLIY
Mus musculus CAM15900	260	GGYYNCQ**D**KYKPQSLPDHKWEMCTSMDRASWGYRKDMTMSTIAKENEIIE
Rattus norvegicus AAH81844	270	GGYYNCE**D**KYRPHSLPDHKWEMCTSVDKASWGYRRDMSMSTIVDENEIIE
***Homo sapiens* (FucA1) P04066**	274	GGYYNCE**D**KFKPQSLPDHKWEMCTSIDKFSWGYRRDMALSDVTEESEIIS
Macaca fascicularis BAD51957	276	GGYYNCE**D**KFKPQSLPDHKWEMCTSIDKFSWGYRRDMAMSDVTEESEIIS
Sus scrofa AEI59128	275	GGYYNCQ**D**KFKPETLPDHKWEMCTSIDKMSWGYRRDMMITDVASEYSIIS
Canis lupus P48300	274	GGYYNCQ**D**KYKPESLPDLKWEMCTSIDKVSWGYRRNMVMSDVASECEIIS
Xenopus laevis AAH42266	268	GGYYNCA**D**KFTPSSLPAHKWEKCTSVDTYSWGYRRNMQVNQLMNERNIIA
Halocynthia roretzi BAB85519	296	GDFRDCN**D**RFTPSKLQTHKWENCMTIDKYSWGFRREANIGDYLTTKELLQ
Phallusia mammillata CAB3247378	263	GGFLTCD**D**RYNPHTLQTRKWENALTIDKHSWGYRRNANFNDFMSINELLQ
Patiria pectinifera BBG92283	271	GGFYSCN**D**RYNPGTLQKHKWENAMTIDKKSWGFRRDAQLDDYLTTDELVA
Danio rerio AAH80244	259	GGYYTCT**D**RYNPGHLVKHKWENCLSIDQRSWGYRREAKLSDYLSTEQLIA
Homo sapiens (FucA2)Q9BTY2	272	GGFYTCS**D**RYNPGHLLPHKWENCMTIDKLSWGYRREAGISDYLTIEELVK
Mus musculus AAH04039	266	GGYYTCS**D**RYNPGYLLPHKWENCMTIDKFSWGYRREAEISDYLTIEELVK
Rattus norvegicus AAH78933	264	GGYYTCS**D**RYNPGHLLPHKWENCMTIDKFSWGYRREAEIGDYLTIEELVK
Staurotypus triporcatus ATP07169	265	GGYYTCS**D**RYNPGHLLPHKWENCMTIDKRSWGYRRNTQLSDYLTIEELVK
Glyptemys insculpta ATP07193	264	GVYYTCS**D**RYNPGHLLPHKWENCMTIDKWSWGYRRNTQLSDYLTIEELVK
Emydura macquarii ATP07217	265	GGYYTCS**D**RYNPGHLLPHKWENCMTIDKRSWGYRSNTQLSDYLTIEELVK
Cyprideis torosa CAD7226149	761	GGYHTCS**D**KYNPGVLQSHKWENCLTVDRRAWGFRRTLRVEEVLTIQELIE
Caenorhabditis elegans CAA91546	254	GGFMTYS**D**HYDPGKLLEKKWENCMTLDKHSWGNRRDMKASEVNTAYEIIE
Ascaris suum ADY44827	265	GGFLTYM**D**HYDPGHLLPRKWESCTTLDRYAWGNRRNMRSSDVLSASEVID
Apis cerana AEY59383	284	GDFYTCS**D**RYNPGILLPHKWENCMTIDRKSWGFRRNAILSEYFTLAELIK

Figure S9. Abbreviated multiple sequence alignment of animal GH29 α-L-fucosidases. Related to Figure 5. Sequences of animal fucosidases were downloaded from the Cazy database and aligned using COBALT. The conserved acid/base residues are labelled with * and highlighted with bold lettering.

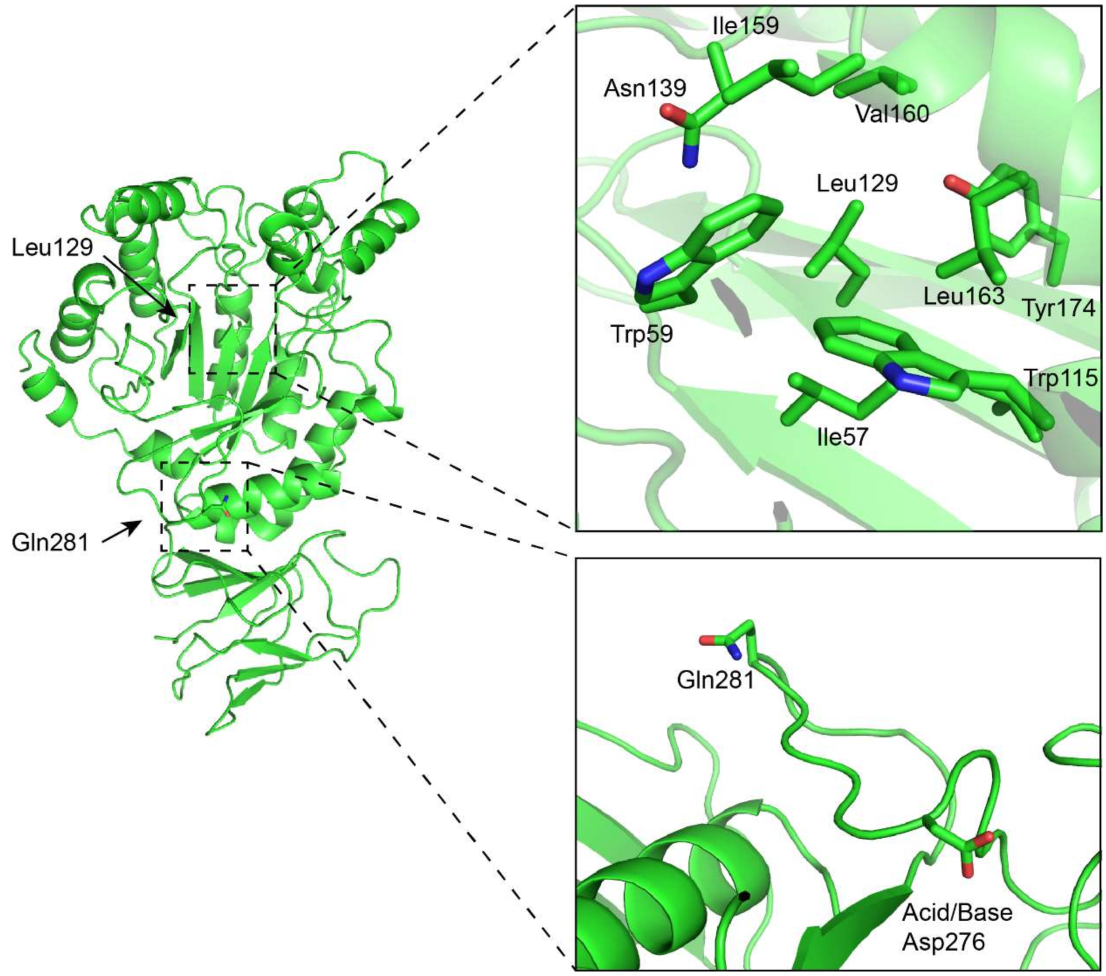

Figure S10. Positions of selected natural variants of FucA1. Related to Figure 6. The local environment of both Leu129 and Gln281 are shown.

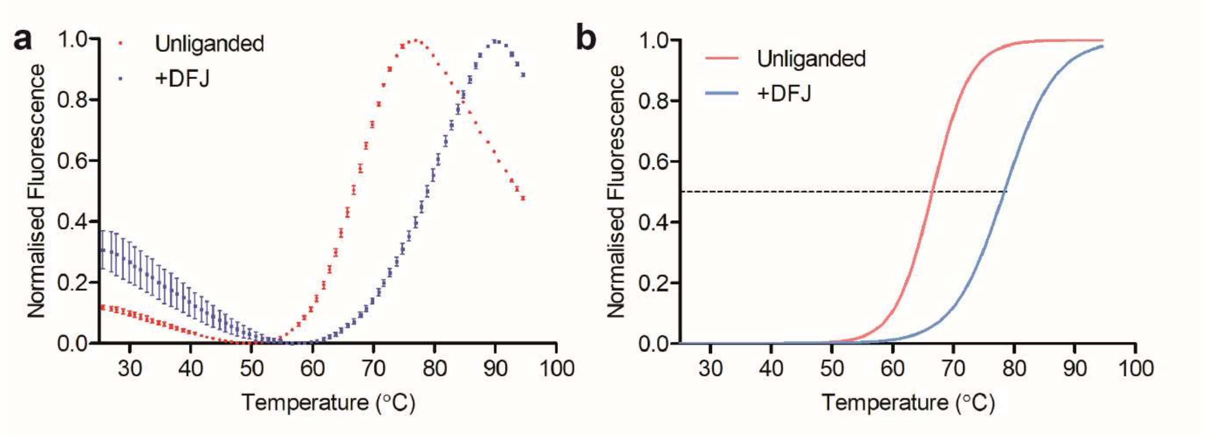

Figure S11. Thermal shift analysis of wild-type FucA1 with and without 25 µM DFJ. Related to Table 4. **a)** Mean of three fluorescence normalised replicates ± standard deviation. **b)** Fit of data to a Boltzmann model. Calculated T_m values are 66.57 ± 0.03 °C for the unliganded FucA1 and 78.4 ± 0.06 °C for the FucA1-DFJ complex.

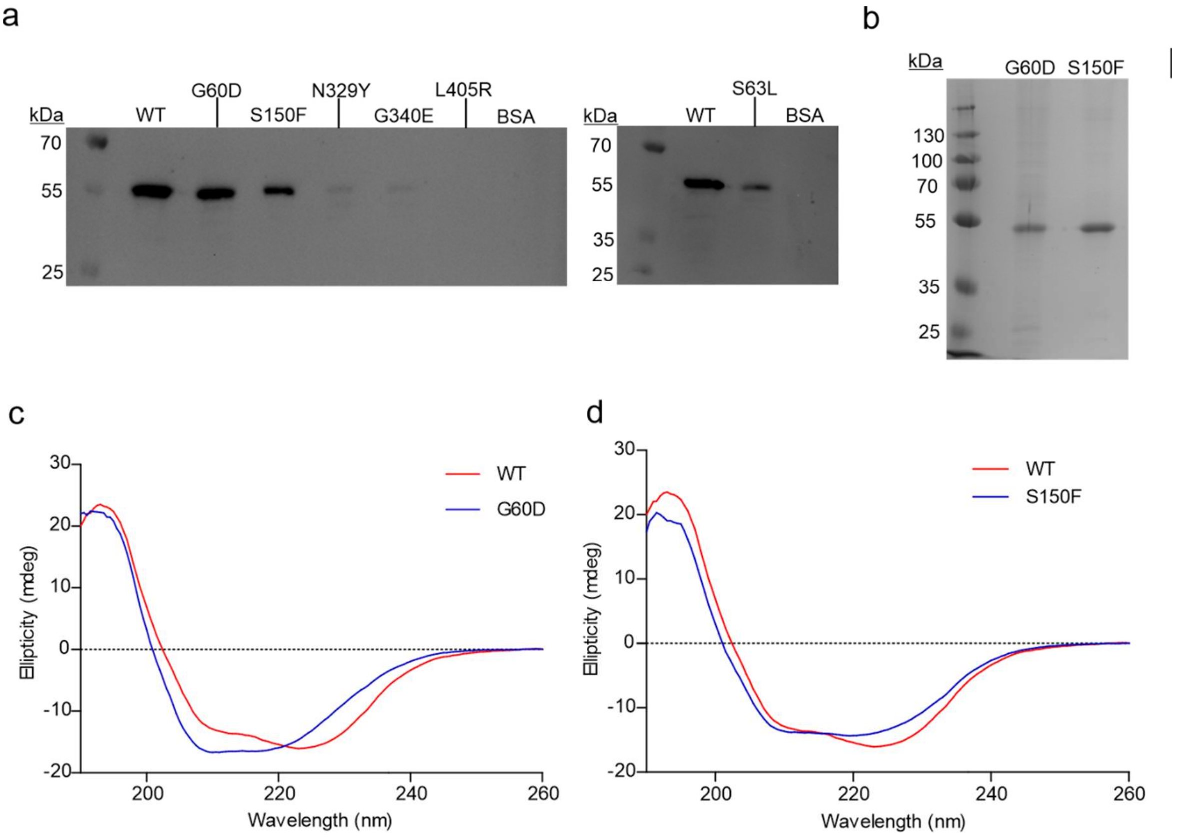

Figure S12. Analysis of FucA1 disease mutants. Related to Table 4. **a)** Western blot of a disease mutant expression trial, 100 ng of BSA was loaded as a control. **b)** SDS-PAGE gel depicting purity of mutant variants. **c)** Comparison of the circular dichroism spectra of Gly60Asp against wild-type FucA1. **d)** Comparison of the circular dichroism spectra of Ser150Phe against wild-type FucA1.

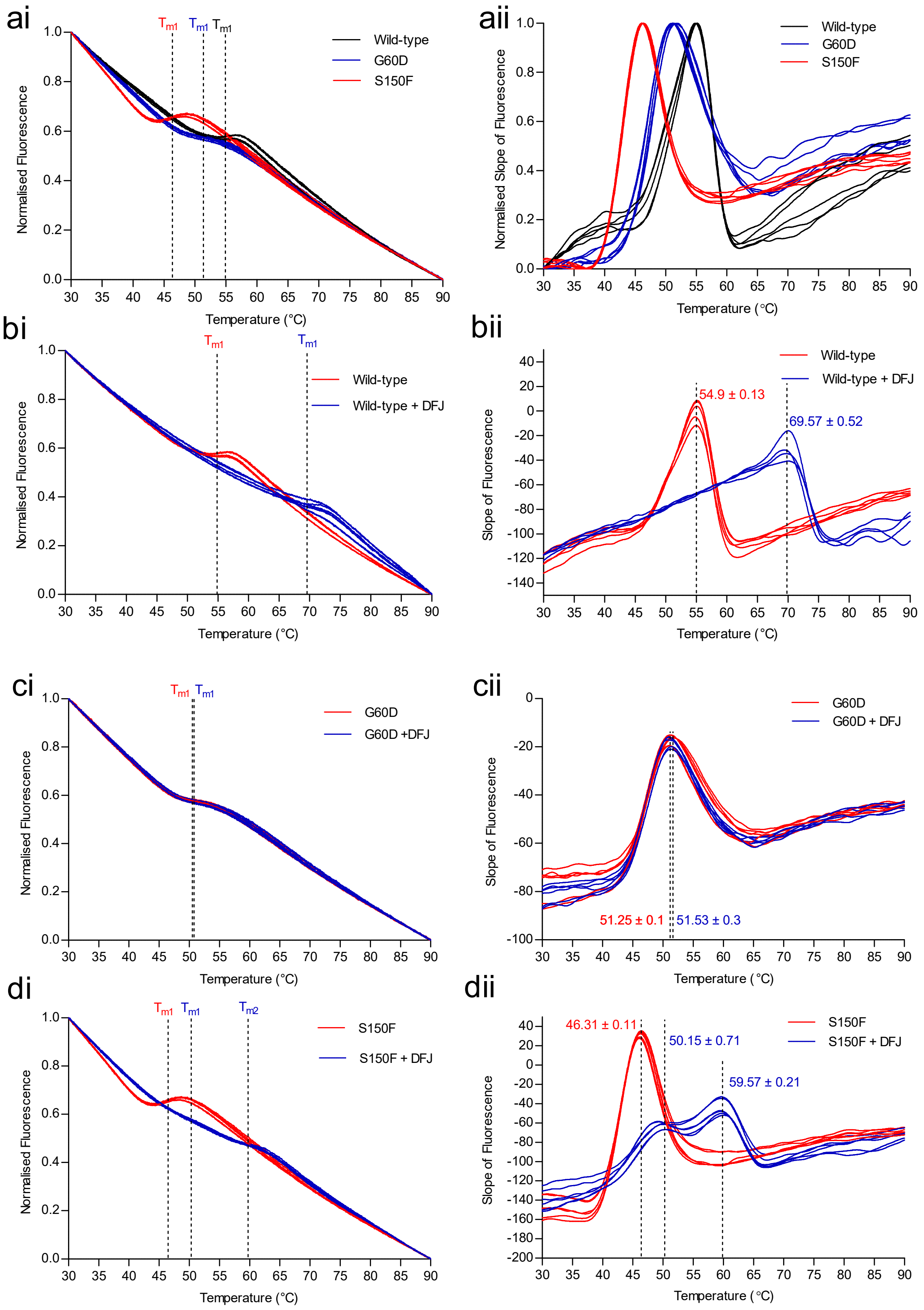

Figure S13. Nano differential scanning fluorimetry of wild-type and disease mutation carrying variants of FucA1. Related to Table 4. Each line represents a technical repeat. **a)** Comparison of normalised 330 nm tryptophan fluorescence of wild-type FucA1 against disease mutants (**ai**) and the

calculated slope of fluorescence (**aii**). **b)** Wild-type protein ± DFJ. **c)** Gly60Asp ± DFJ. **d)** Ser150Phe ± DFJ.
